# Identifying Cases of Type 2 Diabetes in Heterogeneous Data Sources: Strategy from the EMIF Project

**DOI:** 10.1371/journal.pone.0160648

**Published:** 2016-08-31

**Authors:** Giuseppe Roberto, Ingrid Leal, Naveed Sattar, A. Katrina Loomis, Paul Avillach, Peter Egger, Rients van Wijngaarden, David Ansell, Sulev Reisberg, Mari-Liis Tammesoo, Helene Alavere, Alessandro Pasqua, Lars Pedersen, James Cunningham, Lara Tramontan, Miguel A. Mayer, Ron Herings, Preciosa Coloma, Francesco Lapi, Miriam Sturkenboom, Johan van der Lei, Martijn J. Schuemie, Peter Rijnbeek, Rosa Gini

**Affiliations:** 1 Regional Agency for Healthcare Services of Tuscany, Epidemiology unit, Florence, Italy; 2 Department of Medical Informatics, Erasmus University Medical Center, Rotterdam, Netherlands; 3 British Heart Foundation Glasgow Cardiovascular Research Centre, University of Glasgow, Glasgow, United Kingdom; 4 Pfizer Worldwide Research and Development, Groton, Connecticut, United States of America; 5 Department of Biomedical Informatics, Harvard Medical School & Children’s Hospital Informatics Program, Boston Children’s Hospital, Boston, Massachusetts, United States of America; 6 GlaxoSmithKline, Worldwide Epidemiology GSK, Stockley Park West, Uxbridge, United Kingdom; 7 PHARMO Institute for Drug Outcomes Research, Utrecht, Netherlands; 8 The Health Improvement Network, Cegedim Strategic Data Medical Research Ltd, London, United Kingdom; 9 Quretec, Software Technology and Applications Competence Center, University of Tartu, Tartu, Estonia; 10 Estonian Genome Center, University of Tartu, Tartu, Estonia; 11 Tartu University Hospital, Tartu, Estonia; 12 Health Search, Italian College of General Practitioners and Primary Care, Firenze, Italy; 13 Department of Clinical Epidemiology, Aarhus University Hosptial, Aarhus, Denmark; 14 University of Manchester, Manchester, United Kingdom; 15 Arsenàl.IT Consortium, Veneto's Research Centre for eHealth Innovation, Treviso, Italy; 16 Hospital del Mar Medical Research Institute (IMIM) and Universitat Pompeu Fabra, Barcelona, Spain; 17 Janssen Research & Development, Epidemiology, Titusville, New Jersey, United States of America; 18 Observational Health Data Sciences and Informatics, New York, New York, United States of America; Universita degli Studi di Firenze, ITALY

## Abstract

Due to the heterogeneity of existing European sources of observational healthcare data, data source-tailored choices are needed to execute multi-data source, multi-national epidemiological studies. This makes transparent documentation paramount. In this proof-of-concept study, a novel standard data derivation procedure was tested in a set of heterogeneous data sources. Identification of subjects with type 2 diabetes (T2DM) was the test case. We included three primary care data sources (PCDs), three record linkage of administrative and/or registry data sources (RLDs), one hospital and one biobank. Overall, data from 12 million subjects from six European countries were extracted. Based on a shared event definition, sixteeen standard algorithms (*components)* useful to identify T2DM cases were generated through a top-down/bottom-up iterative approach. Each component was based on one single data domain among diagnoses, drugs, diagnostic test utilization and laboratory results. Diagnoses-based components were subclassified considering the healthcare setting (primary, secondary, inpatient care). The Unified Medical Language System was used for semantic harmonization within data domains. Individual components were extracted and proportion of population identified was compared across data sources. Drug-based components performed similarly in RLDs and PCDs, unlike diagnoses-based components. Using components as building blocks, logical combinations with AND, OR, AND NOT were tested and local experts recommended their preferred data source-tailored combination. The population identified per data sources by resulting algorithms varied from 3.5% to 15.7%, however, age-specific results were fairly comparable. The impact of individual components was assessed: diagnoses-based components identified the majority of cases in PCDs (93–100%), while drug-based components were the main contributors in RLDs (81–100%). The proposed data derivation procedure allowed the generation of data source-tailored case-finding algorithms in a standardized fashion, facilitated transparent documentation of the process and benchmarking of data sources, and provided bases for interpretation of possible inter-data source inconsistency of findings in future studies.

## Introduction

In recent years, an increasing number of projects have been focusing on re-using existing electronic health records (EHR) for clinical research.[1] In particular, huge efforts have been made to combine health data from isolated environments and perform valid multi-data source observational studies.[2, 3]

In this context, the European Medical Information Framework (EMIF) project was launched with the main objective of building an infrastructure for the efficient re-use of existing European health care data for epidemiological research (http://www.emif.eu/). Within the project, a federation of heterogeneous sources of real world data (e.g. administrative, hospital or primary care databases, disease registries, biobanks), currently collecting health information on around 52 million European citizens, collaborate in the EMIF-Platform whose focus is the consistent exploitation of currently available patient-level data to support novel research. One of the main challenges for the EMIF-Platform is to deal with the heterogeneous characteristics of the participating data sources and facilitate the execution of high quality multi-national, multi-data source observational studies based on populations with otherwise unconceivable sample sizes and follow-up time span.

In general, different strategies can be adopted to identify a population of interest from a single source of EHR.[4, 5] The choice of a particular case-finding algorithm is generally driven by both the specific research question and the data source peculiarities.[6] The chosen algorithm, however, can significantly affect the characteristics of the cases identified [4, 5] and, for this reason, should be carefully taken into account when discussing study results.

In multi-data source studies, tailored choices may be necessary [6–8], and the diversity of local case-identification algorithms may increase along with the heterogeneity of the data sources involved [6, 9, 10]. A transparent process of documentation and evaluation of local case-finding algorithms becomes paramount for the correct interpretation of study results as well as for the discussion of possible inter-data source inconsistency of study findings [10–12]. It must be noted that data sources available to study European populations are much more heterogeneous than data sources from a single country, such as the United States [10]. Therefore, in order to address this issue, the EMIF-Platform designed a novel standard procedure for data derivation which leverages the experience gained from previous European multi-national, multi-data source studies [2, 3, 9, 13]. In this proof-of-concept study, the identification of type 2 diabetes mellitus (T2DM), a common chronic condition with important implications for future health[14], was used as a test case.

## Materials and Methods

### Data sources

Eight European data sources collecting health care information on around 20 million subjects from six different countries participated to this study (Table 1). Three were primary care data sources (PCDs), three were record linkage systems of different registries (RLDs), one was a hospital data source (HD) and one was a biobank (BD). In specific, the three primary care data sources were the Health Search IMS Health LPD database (HSD, Italy),[9, 15] the Integrated Primary Care Information database (IPCI, The Netherlands)[16] and The Health Improvement Network database (THIN, UK), in which the general practitioners (GPs) function as data keeper of all patient’s medical information.[17] The three record linkage data sources were the Aarhus University Hospital (AUH, Aarhus, Denmark),[18, 19] PHARMO (PHARMO, The Netherlands)[20] and the Regional Health Authority of Tuscany (ARS, Italy),[9, 15] which collect data from different sources (e.g. hospital discharge records, death registries, drug dispensing and procedures). The HD was the Information System of Parc de Salut Mar Barcelona (IMASIS, Spain) that records information from routine healthcare activities of Hospital del Mar of Barcelona.[21, 22] The BD was the Estonian Genome Center of University of Tartu (EGCUT, Estonia) in which information from interviews of voluntary donors of biological samples is collected through standard questionnaires.[23] EGCUT is the only cross-sectional data source included in this study. In all data sources except the Spanish HD, IMASIS, information on a representative sample of the population living in the corresponding geographic area are collected. In the Italian PCD and in the Estonian BD only adult population is represented (>14 and >18 years of age, respectively). The information in the corresponding databases is recorded using different coding systems. Diagnoses are coded according to the International Classification of Diseases, 9th Revision, Clinical Modification (ICD-9-CM) or ICD-10 (10th version), the International Classification of Primary Care (ICPC), READ or are as free text. Prescriptions/dispensings are coded according to the Anatomical Therapeutic Chemical classification (ATC) or BNF/Multilex. The majority of the data sources collect records concerning the utilization of diagnostic procedures and laboratory results. The coding of these data domains are based on local service terminologies.

**Table 1:** Data sources’ characteristics*.

Data source (Original organizationacronym)	Type of data source	Catchment area	Cumulative number of participants in the database	Average follow-up time	Diagnoses • setting, • coding system	Medication (coding system)	Diagnostic procedures/tests (coding system)	Laboratory results (coding system for measurments)
**RLD-I** (ARS)	Record linkage system	Tuscany (Italy)	5 millions	9 years	• Inpatient • ICD9CM	ATC	ICD9CM or local terminology	-
**RLD-DK** (AUH)	Record linkage system	The northern and central region of Jutland. (Denmark)	2.3 millions	13 years	• Inpatient, secondary care • ICD10	ATC	NOMESCO	-
**RLD-N** (PHARMO)	Record linkage system	Netherlands (Certain regions, mainly South East and North-West)	10 millions	10 years	• Inpatient • ICD9CM	ATC	Local terminology	Local terminology
**PCD-I** (HSD)	Primary care	Italy	2.3 millions	10 years	• Primary care • ICD9CM	ATC	Local terminology	Local terminology
**PCD-UK** (THIN)	Primary care	United Kingdom	12 millions	9 years	• Primary care, READ	ATC	Local terminology	Local terminology
**PCD-N** (IPCI)	Primary care	Netherlands	2.8 millions	3 years	• Primary care • ICPC/free text	ATC	Local terminology	Local terminology
**HD** (IMASIS)	Hospital	Barcelona (three city districts)	1.5 millions	5 years	• Admissions, outpatients, major ambulatory surgery and emergency room • ICD9CM	Local terminology & the Spanish Medicines Agency codes	ICD9CM	Local terminology
**BD** (EGCUT)	Biobank	Estonia	52000	Not applicable	• Primary care/Self reported • ICD10	ATC	Local terminology	Local terminology

*Information reported in the table is updated at January 2013.

### Study population and design

In each participating data source the study population corresponded to all active subjects on January the 1^st^ 2012 (reference date) that at the same date had ≥16 years of age. Due to sample size issues, exception was made for EGCUT in which January the 1^st^ 2009 was considered as the reference date.

A descriptive, cross-sectional, retrospective multi-database study was performed. Patients with T2DM were identified within the populations selected from the participating data sources by using different case-finding algorithms.

### Event definition

T2DM is a chronic clinical condition characterized by hyperglycemia due to insulin resistance and a progressive deficiency in insulin production[14]. It represents the most common form of diabetes, comprising about 90% of all cases of diabetes worldwide[24]. Diagnosis and follow-up of T2DM is based on laboratory tests for blood glucose measurements and treatment includes life style interventions (i.e. diet and physical exercise) and use of medications[14].

**Fig 1:**
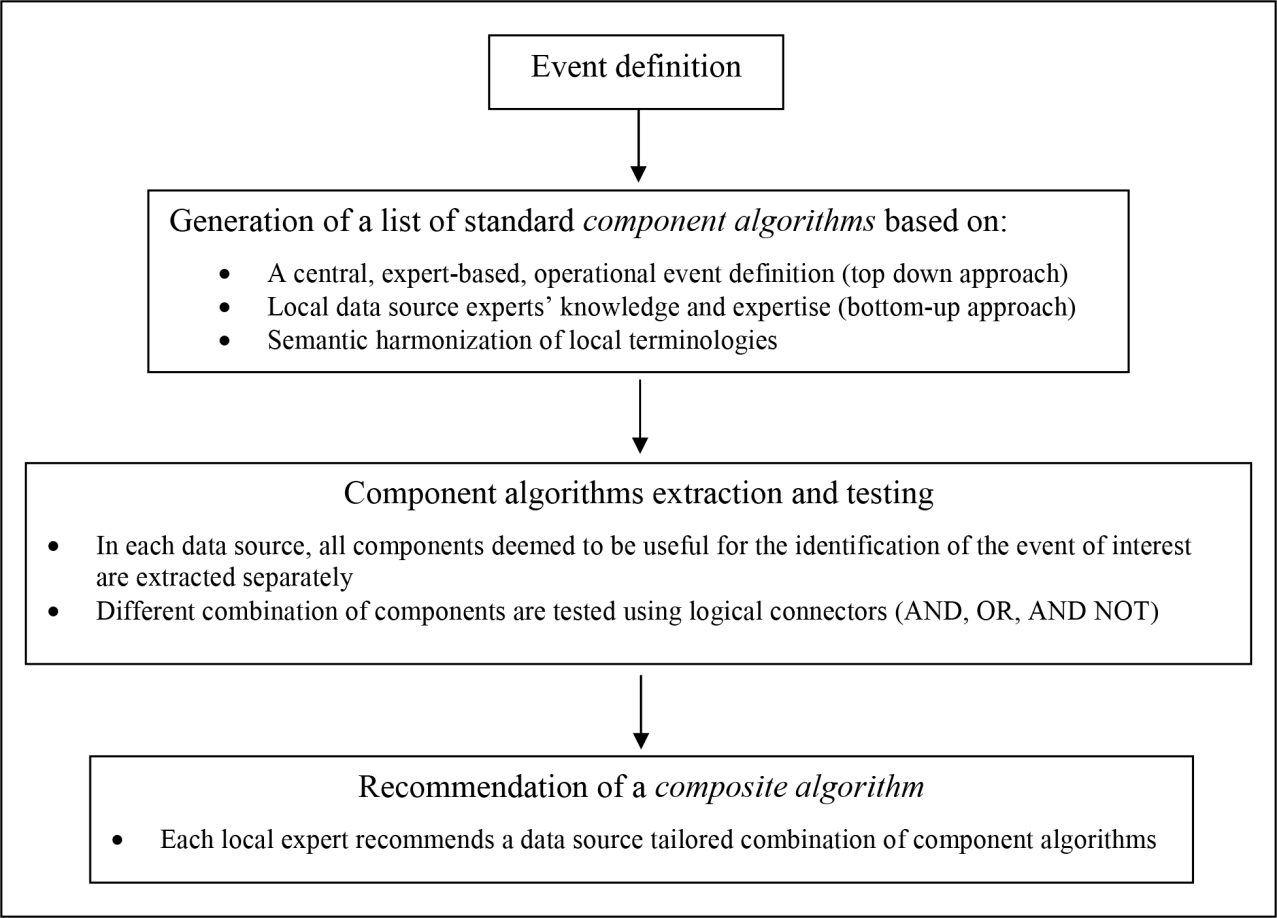
The standard procedure for data derivation.

As the first step of the data derivation procedure, a shared clinical definition of T2DM was adopted (Fig 1) and defined according to the European Society of Cardiology and European Association for the Study of Diabetes (ESC/EASD) guideline[14].

### Generation of a list of *component algorithms*

To identify subjects with T2DM in a healthcare data source, information from one or more data domains may be available. Diagnoses and/or records collecting information on routine patients’ clinical care and follow-up, such as drug prescriptions, utilization of diagnostic tests and laboratory results, can be used,[4, 25, 26] so that combining data from one or more of these domains, different case-finding strategies, with different sensitivity and positive predictive value (PPV), can be obtained.[4]

As the second step of the data derivation procedure a unique list of standard algorithms useful to identify cases of T2DM in the selected data sources was generated. Such standard algorithms, referred to as “component algorithms”, were defined as rules to identify subjects with a defined pattern of records selected from a single data domain. For the identification of T2DM, a total of four data domains were concerned: diagnoses (DIAG), drug prescription/dispensing (DRUG), utilization of a diagnostic test (TEST) or laboratory results (LABVAL). Component algorithm could be intended as inclusion, exclusion or refinement criteria. Two sources of knowledge were leveraged and integrated for the design of the component algorithms: a central expert-based definition of T2DM (top-down engineering) and the expertise provided by local data source experts (bottom-up learning).[8] The top-down engineering was embedded in the previously mentioned clinical definition (see previous subsection) and in an *operational* definition. The latter was intended as a description of typical diagnostic and therapeutic patterns that patients with T2DM are expected to follow. Both the *clinical* and *operational definition* were created by researchers with clinical expertise and agreed with both the local data source experts and the central study leader. The bottom-up learning, instead, was embedded in a questionnaire where local experts were asked to describe the algorithms they would have used to identify T2DM cases in their own data source, possibly mentioning relevant validation studies. All the information gathered were then used by the central study leader to create a unique list of inclusion, exclusion and refinement criteria corresponding to those mentioned at least once in one of the documents generated. The list was created on the grounds of the central study leaders' judgement and revised by the clinical and local experts. Each criterion was then translated into a standard component algorithm, as follow. As already described in greater details in a previous published paper,[3] the Unified Medical Language System (UMLS) was used to build a shared semantic foundation across the different coding systems: medical concepts pertinent to the clinical and operational definition of T2DM were identified and projected to local terminologies. The final list of local codes, strings and free text keywords was obtained through an iterative process involving local experts’ feedback. Each component algorithm was fully described by two additional rules: the first was the pattern of records that triggers identification of the event (for instance: at least two records in the same calendar year), and the second concerned the criteria to identify the case’s index date (e.g. date of the first record).

### Data extraction and analysis: “*the component algorithm strategy*”

A distributed network approach was adopted in EMIF to allow partners for maintaining control of their data and to benefit from local data source experts consultation on the appropriate use of data and interpretation of results.[2, 3] Local experts were asked to select and extract all component algorithms considered useful to identify T2DM cases in their data source. All person-time available up to the reference date was considered for algorithm application. Extracted data were prepared to be inputted in Jerboa, a custom-built software developed in the EU-ADR project[2] which was run locally to standardize the data aggregation process. After providing formal approval, local data source experts uploaded aggregated analytical datasets to a common virtual machine.

Using a custom-built analysis tool (a Microsoft Access interface for Stata [StataCorp. 2013. Stata Statistical Software: Release 13. College Station, TX: StataCorp LP] and LaTeX [https://www.latex-project.org/]), local experts could test the extracted components in any possible logical combinations by using Boolean operators (i.e. AND, OR, AND NOT). This strategy, we referred to as *“the component algorithm strategy”*, allowed local experts to build more complex case-finding strategies (*composite algorithms)* by combining two or more of the extracted components as a mean of inclusion, exclusion or refinement criteria.The process of testing different combinations of components was led separately by each local expert, who finally chose a particular combination of components as the *recommended composite algorithm* for the identification of T2DM in the relevant data source. The local experts, the clinical experts and the central study leader held a series of conference calls to discuss the final choice about the recommended composite algorithm, but in case of disagreement the local expert opinion prevailed. A comment describing the reasons behind the choice was recorded together with an estimate, either objective or subjective, of the expected sensitivity and PPV. This information, as well as the minutes from the conference calls, was stored and intended as a source of reusable knowledge.

### Presentation of results

Results from the application of individual components and recommended composite algorithms were compared across data sources and presented as age-specific percentages of subjects identified in the study population of the corresponding data source.

In each participating data source, the impact of extracted component was assessed with respect to the total number of subjects identified using the recommended composite algorithm, which was considered as the *reference case population*. For this purpose, we calculated: i) the percentage of subjects identified by each component in the reference case population and ii) the prevalence rate ratio (PRR) of subjects identified by the recommended composite algorithm with and without the use of the tested component as additional inclusion criteria, i.e. PRR = ((N in tested component OR in recommended composite algorithm)/ N in recommended composite algorithm)-1.

Patient records were anonymized and de-identified prior to analysis and only aggregated data were shared across sites therefore no written informed consent was necessary for this study. Permission for both re-use of the data analyzed in this study as well as for publication of the results obtained was granted by each participating organizations’ review board.

The full protocol of the research project is publicly available on the electronic register of observational studies of the European Network of Centers for Pharmacoepidemiology and Pharmacovigilance (http://www.encepp.eu/encepp/viewResource.htm?id=11158).

## Results

Since this was a proof-of-concept study, results presented here are not intended as estimates of disease frequency.

Overall, the EMIF-Platform provided for this study aggregated health data from around 12 million European citizens.

The size of the study populations selected from the participating data sources ranged from 1600 to 3.4 million subjects. Components algorithms included in at least one recommended composite algorithms are reported in Table 2.

**Table 2:** Component algorithms description.

Component algorithm acronym	Algorithm description	Record retrieval rules*	Case’s index date
DIAG_T2DM_PC	Patients who have ≥1 diagnoses of T2DM recorded in a primary care setting	Records of (Diabetes type 2) occurs in [diagnosis fields] of [tables collected during primary care]	1^st^ record
DIAG_T2DM _SC	Patients who have ≥1 diagnoses recorded in a secondary care setting	Records of (Diabetes type 2) occurs in [diagnosis fields] of [tables collected during secondary care]	1^st^ record
DIAG_T2DM _INP	Patients who ≥1 diagnoses recorded during a hospital admission	Records of (Diabetes type 2) occurs in [diagnosis fields] of [tables collected during inpatient care]	1^st^ record
DIAG_DMUNSPEC	Patients who ≥1 diagnoses of unspecified diabetes recorded in primary, secondary, or inpatients care	Records of (Diabetes unspecified) occurs in [diagnosis fields] of [tables collected in primary, secondary, or inpatients care]	1^st^ record
DIAG_DMUNSPEC_OTH	Patients who have ≥1 diagnoses recorded in a setting other than primary, secondary, or inpatients care	Records of (Unspecified diabetes) occurs in [diagnosis fields] of [tables collected in other settings]	1^st^ record
DIAG_T1DM	Patients who have ≥1 diagnoses of T1DM recorded in any care setting	Records of (Diabetes mellitus type I) occurs in [diagnosis fields] of [any table collecting diagnoses]	1^st^ record
DIAG_EXCL	Patients who have ≥1 diagnoses of conditions excluding T2DM other than T1DM recorded in any care setting	Records of ((Metabolic problems around pregnancy) OR (Metabolic/pancreatic problems, non type 2 diabetes) OR (Polycystic Ovary Syndrome) occurs in [diagnosis fields] of [any table collecting diagnoses]	1^st^ record
DRUG_INSULIN_ONE	Patients who have ≥1 recorded prescriptions/dispensings of insulin	Records of (Insulins and analogues) occurs in [ATC field] of [drugs tables]	1^st^ record
DRUG_INSULIN	Patients who have ≥2 recorded prescriptions/dispensings of insulin in a calendar year	Records of (Insulins and analogues) occurs in [ATC field] of [drugs tables]	2^nd^ record
DRUG_ORAL_ONE	Patients who have ≥1 recorded prescriptions/dispensings of non-insulin antidiabetic drugs	Records of (Drugs used in diabetes, excl insulin) occurs in [ATC field] of [drugs tables]	1^st^ record
DRUG_ORAL	Patients who ≥2 prescriptions/dispensings of non-insulin antidiabetics in a calendar year	Records of (Drugs used in diabetes, excl insulin) occurs in [ATC field] of [drugs tables]	2^nd^ record
TEST_GLUCO5_1YR	Patients who have ≥5 records of utilization of blood glucose measurements within 1 year	Records of (Blood glucose measurement) occurs in [code of test field] of [tables collecting laboratory test results or dispensings]	5^th^ record
TEST_GLUCO2_PYEAR_5YRS	Patients who have ≥2 records of utilization of blood glucose measurements per year for 5 consecutive years	Records of (Blood glucose measurement) occurs in [code of test field] of [tables collecting laboratory test results or dispensings]	2^nd^ record
LABVAL_ HbA1c	Patients who have ≥2 laboratory results recorded from a glycated hemoglobin test higher than 6.5% (48 mmol/mol)	Records of (Glycated Haemoglobin) occurs in [code of test field] of [tables collecting laboratory test results] AND [result field] of the same record is higher than 6.5% (or 48 mmol/mol, according to unit of measurement adopted in the table)	2nd record
LABVAL_FAST_GLUC	Patients who have ≥2 laboratory results recorded from a fasting plasma glucose measurement higher than 126 mg/dl)	Records of (Fast gluc) occurs in [code of test field] of [tables collecting laboratory test results] AND [result field] of the same record is higher than 126 mg/dl	2nd record
LABVAL_LCURVE_GLUC	Patients who have ≥2 laboratory results recorded from a glucose tolerance test higher than 200 mg/dl	Records of (LcurveGLuc) occurs in [code of test field] of [tables collecting laboratory test results] AND [result field] of the same record is higher than 200 mg/dl	2nd record

*Codes and free text keywords corresponding to the medical concepts embedded in component algorithms (in brackets) are reported in S1 Table.

**Fig 2:**
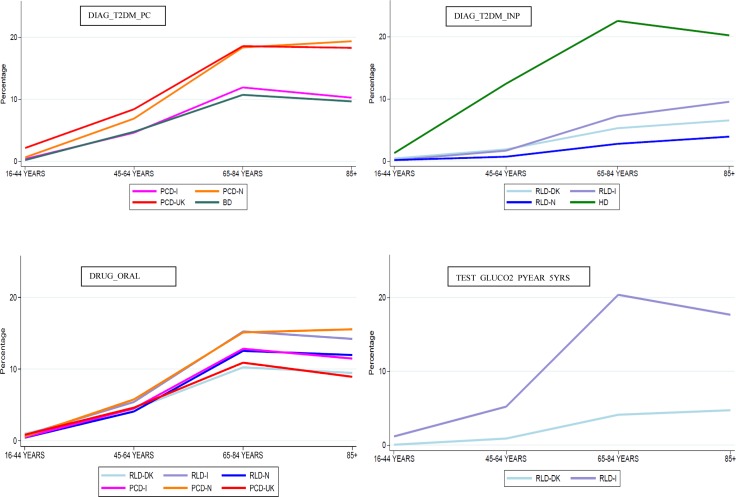
Comparison of results from individual component algorithms: four examples.

In Fig 2 four examples of comparisons of age band-specific results from individual component algorithms across data sources are shown. The full list of comparisons concerning all those components extracted from at least two data sources and included in at least one recommended composite algorithm are available as supporting information in S1 Fig. As for DIAG-based components very different performances were associated to the healthcare setting of data collection (primary, secondary, inpatient care). The component DRUG_ORAL (i.e. ≥2 records of non-insulin antidiabetic drugs utilization in one calendar year) and DRUG_INSULIN (i.e. ≥2 records of insulin utilization in one calendar year) were extracted in all the participating PCDs and RLDs and resulted in a comparable age band-specific percentage of subjects identified in the respective study populations.

**Fig 3:**
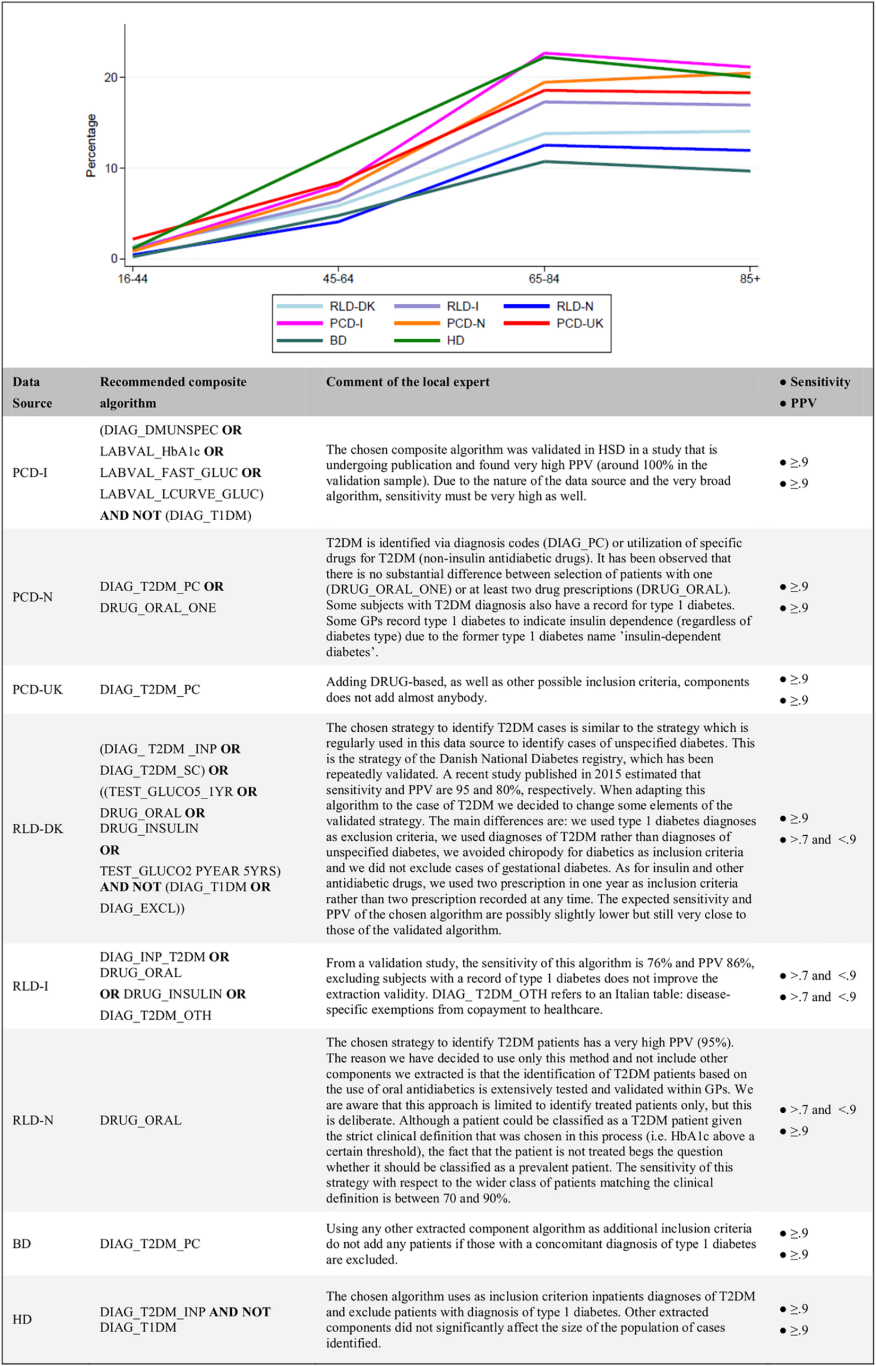
Recommended composite algorithms: age band-specific percentages of subjects identified on the relevant total study population. PPV: Positive Predictive Value.

The data source tailored recommended composite algorithms are shown in Fig 3 together with the comments of the local experts. The PCD from UK and the BD from Estonia adopted the component algorithm based on T2DM diagnoses from primary care (DIAG_T2DM_PC) only as the recommended choice. The HD from Spain excluded from the pool of subjects identified through inpatients diagnoses of T2DM (DIAG_T2DM_INP) those with a recorded diagnosis of type 1 diabetes (DIAG_T1DM). Only three data sources (the PCD from Italy and the RLDs from Denmark and Italy) had previous validation studies available [25, 27, 28]. They all adopted a final composite algorithm based on the relevant validation study.

The Dutch PCD added a sensitive pattern of utilization of non-insulin antidiabetic drugs (i.e. DRUG_ORAL_ONE) as inclusion criterion, due to the observed low sensitivity of the DIAG-based algorithm DIAG_T2DM_PC in this data source. The Dutch RLD chose to include only subjects utilizing non-insulin antidiabetics, because the available DIAG-based component that used diagnoses from inpatients setting was considered unreliable by local experts.

Through the application of the recommended composite algorithm, the lowest percentage of study population was identified in the Estonian BD, 3.5%, while the highest in the Spanish HD, 15.7%. In the RLDs it ranged from 4.1% to 7.5% while in PCDs from 6.8% to 8.6%. The age band-specific percentages of the total case populations identified using the recommended composite algorithms showed more comparable results across all participating data sources (Fig 3). The expected sensitivity of the recommended composite algorithms, as reported by local experts either from previous validation studies or from subjective judgement, was >0.9 in all data sources except for the Italian and Dutch RLDs for which a sensitivity between 0.7 and 0.9 was expected. As for PPV, the Italian and Danish RLDs only reported an expected value ranging from 0.7 and 0.9 while for the remaining data sources the Fig was >0.9.

The union of any extracted DIAG-based component among the five intended as inclusion criteria identified from 93 to 100% of the reference case population in PCDs (Table 3), 100% in both BD and HD, and from 15% to 73% in RLDs. In RLDs, DRUG-based components identified from 81% to 100% of the respective total case population, while from 58% to 83% in PCDs. TEST-based components were included in the recommended composite algorithm of the Danish RLD only in which these algorithms identified 44% of the total case population. Although TEST-based components were also extracted from the Italian RLD, they were not included in the recommended composite algorithm since they would have almost doubled the total case population (PRR = +79.2%), thus suggesting a too low specificity. LABVAL-based algorithms were included in the recommended composite algorithm of the Italian PCD only: overall, the three components from this data domain identified 46% of the total case population. Notably, subjects from the same data source could be identified by one or more component thus the percentages reported above may overlap.

**Table 3:** Impact of extracted component algorithms on total case population identified in each participating data source through the application of the relevant recommended composite algorithm.

**COMPONENT ALGORITHMS (B)°**		**RECOMMENDED COMPOSITE ALGORITHMS (A)**							
		**RLD-I**	**RLD-DK**	**RLD-N**	**PCD-UK**	**PCD-N**	**PCD-I**	**BD**	**HD**
	N	3391177	1372883	1405220	3278013	992924	945691	22430	15713
	N in A	254045	77616	57712	253197	67096	81658	779	2466
	% of A in N	7.5	5.7	4.1	7.7	6.8	8.6	3.5	15.7
**DIAG_T2DM_PC** (≥1 diagnosis from primary care)	N in B	n.e.	n.e.	n.e.	253197	62191	43438	779	n.e.
	% of B in A	-	-	-	100.0%	92.7%	52.6%	100.0%	-
	PRR if B added	-	-	-	+0.0%	+0.0%	+0.6%	+0.0%	-
**DIAG_T2DM_INP** (≥1 T2DM diagnosis from inpatient care)	N in B	95303	27887	13098	n.e.	n.e.	n.e.	n.e.	2520
	% of B in A	37.5%	35.9%	15.1%	-	-	-	-	100.0%
	PRR if B added	+0.0%	+0.0%	+7.6%	-	-	-	-	+2.2%
**DIAG_T2DM_SC** (≥1 T2DM diagnosis from secondary care)	N in B	n.e.	35744	n.e.	n.e.	n.e.	n.e.	n.e.	n.e.
	% of B in A	-	46.1%	-	-	-	-	-	-
	PRR if B added	-	+0.0%	-	-	-	-	-	-
**DIAG_DMUNSPEC** (≥1 unspecified diabetes diagnosis from any healthcare setting)	N in B	191999	n.e.	n.e.	n.e.	n.e.	79035	n.e.	n.e.
	% of B in A	73.2%	-	-	-	-	94.3%	-	-
	PRR if B added	+2.4%	-	-	-	-	+2.5%	-	-
**DIAG_ DMUNSPEC_OTH** (≥1 unspecified diabetes diagnosis from co-payment exemption)	N in B	149806	n.e.	n.e.	n.e.	n.e.	n.e.	n.e.	n.e.
	% of B in A	59.0%	-	-	-	-	-	-	-
	PRR if B added	+0.0%	-	-	-	-	-	-	-
**DIAG_T1DM** (≥1 type 1 diabetes diagnoses from any healthcare setting)	N in B	18147	17896	n.e.	n.e.	8816	2050	164	78
	% of B in A	6.9%	18.1%	-	-	8.8%	0.0%	2.8%	0.0%
	PRR if B added	+0.2%	+4.9%	-	-	+4.3%	+2.5%	+18.2%	+3.2%
**DIAG_EXCL** (≥1 diagnoses of other types of diabetes or glucose intolerance)	N in B	13741	7895	2904	n.e.	n.e.	5782	n.e.	78
	% of B in A	1.1%	1.8%	1.5%	-	-	0.3%	-	1.7%
	PRR if B added	+4.3%	+8.3%	+3.5%	-	-	+6.8%	-	+1.5%
**DIAG_T2DM_PC** OR **DIAG_T2DM_INP** OR **DIAG_T2DM_SC** OR **DIAG_DMUNSPEC** OR **DIAG_DMUNSPEC_OTH**	N in B	191999	43622	13098	253197	62191	79035	779	2520
	% of B in A	73.2%	56.2%	15.1%	100.0%	92.7%	94.3%	100.0%	100.0%
	PRR if B added	+2.4%	+0.0%	+7.6%	+0.0%	+0.0%	+2.5%	+0.0%	+2.2%
**DRUG_INSULIN** (≥2 prescriptions/dispensings of insulin in one calendar year)	N in B	45522	22074	21192	41019	15020	11607	n.e.	n.e.
	% of B in A	17.9%	25.4%	25.8%	16.1%	19.0%	12.3%	-	-
	PRR if B added	+0.0%	+3.0%	+10.9%	+0.1%	+3.4%	+2.0%	-	-
**DRUG_INSULIN_ONE** (≥1 prescriptions/dispensings of insulin)	N in B	62341	23319	0	0	17719	0	18	0
	% of B in A	21.2%	26.5%	-	-	22.0%	-	1.5%	-
	PRR if B added	+3.4%	+3.6%	-	-	+4.4%	-	+0.8%	-
**DRUG_ORAL** (≥2 prescriptions/dispensings of NIAD in one calendar year)	N in B	216338	57153	57712	136370	51589	45624	-	0
	% of B in A	85.2%	71.0%	100.0%	51.7%	76.9%	53.0%	-	-
	PRR if B added	+0.0%	+2.7%	+0.0%	+2.1%	+0.0%	+2.9%	-	-
**DRUG_ORAL_ONE** (≥1 prescriptions/dispensings of NIAD)	N in B	273952	61604	0	0	54181	62110	45	0
	% of B in A	87.5%	72.7%	-	-	80.8%	70.6%	5.8%	-
	PRR if B added	+20.3%	+6.7%	-	-	+0.0%	+5.4%	+0.0%	-
**DRUG_INSULIN** OR **DRUG_INSULIN_ONE** OR **DRUG_ORAL** OR **DRUG_ORAL_ONE**	N in B	295676	70405	64016	151576	58355	65076	40	0
	% of B in A	93.0%	81.1%	100.0%	57.7%	82.6%	73.1%	50.0%	-
	PRR if B added	+23.4%	+9.6%	+10.9%	+2.2%	+4.4%	+6.6%	+4.1%	-
**TEST_TEST_GLUCO5_1YR** (≥5 glycated hemoglobin tests in 1 year)	N in B	266940	16999	0	0	0	0	0	0
	% of B in A	45.8%	21.6%	-	-	-	-	-	-
	PRR if B added	+59.3%	+0.3%	-	-	-	-	-	-
**TEST_GLUCO2_PYEAR_5YRS** (≥2 glycated hemoglobin testsper year during 5 consecutive years)	N in B	172784	28583	0	0	0	0	0	0
	% of B in A	32.6%	36.1%	-	-	-	-	-	-
	PRR if B added	+35.4%	+0.7%	-	-	-	-	-	-
**TEST_GLUCO5_1YR** OR **TEST_GLUCO2_PYEAR_5YRS**	N in B	335466	34801	0	0	0	0	0	0
	% of B in A	52.8%	44.1%	-	-	-	-	-	-
	PRR if B added	+79.2%	+0.8%	-	-	-	-	-	-
**LABVAL_FAST_GLUC** (≥2 fasting glucose values >126mg/dl)	N in B	0	0	0	0	0	32153	0	0
	% of B in A	-	-	-	-	-	38.6%	-	-
	PR if B added	-	-	-	-	-	+0.8%	-	-
**LABVAL_HbA1c** (≥2 glycated hemoglobin value >6.5%)	N in B	0	0	62400	0	44271	20196	0	0
	% of B in A	-	-	65.1%	-	63.6%	24.1%	-	-
	PRR if B added	-	-	+43.0%	-	+2.4%	+0.7%	-	-
**LABVAL_LCURVE_GLUC** (≥2 glucose tolerance test values >200mg/dl)	N in B	0	0	0	0	0	32	0	0
	% of B in A	-	-	-	-	-	0.0%	-	-
	PRR if B added	-	-	-	-	-	+0.0%	-	-
**LABAL_FAST_GLUC** OR **LABAL_HbA1c** OR **LABAL_LCURVE_GLUC**	N in B	0	0	62400	0	44271	38764	0	0
	% of B in A	-	-	65.1%	-	63.6%	46.5	-	-
	PRR if B added	-	-	+43.0%	-	+2.4%	+1.0%	-	-

Since patients can be identified by more than one component algorithms, percentages may overlap.

*Grey cells* correspond to component algorithms that were included in the relevant recommended composite algorithm.

NIAD: Non-Insulin Antidiabetic Drugs.

A = recommended composite algorithm.

B = tested component algorithm(s).

N = Study population.

PRR = prevalence rate ratio of “A or B” in N with respect to the percentage of A in N.

n.e. = not extracted

## Discussion

Through the application of the standard data derivation procedure tested in this study, cases of T2DM were identified in eight distinct sources of health data with heterogeneous characteristics. Logical combinations of standardized component algorithms, each based on a single data domain, were used to build data source-tailored case-finding algorithms. This “component algorithm strategy” facilitated both benchmarking and interpretation of results across data sources. It also allowed the assessment of the impact of individual standardized component algorithms on the total population of cases retrieved in each participating data source that ultimately provided insight into the strengths and limitations of each data source with respect to the identification of T2DM cases.

Compared to previous projects that aimed to combine different European sources of EHR for research purposes,[2, 3] the main innovation of the standard procedure tested in this study was the use of component algorithms as building blocks that could be combined to create more complex case-finding algorithms. As demonstrated by the results presented here, in the context of a multi-national, multi-data source study, the “component algorithm strategy” represents an extremely flexible approach for generating EHR-driven[6] case-finding algorithms in a standardized fashion: on the one hand, it allows the local experts’ knowledge of the EHR “natural system”[8] to be fully leveraged, avoiding loss of information and assuring the correctness of the derived information, while, on the other hand, it facilitates the interpretation and benchmarking of results obtained even across data sources with very different characteristics. Notably, the data derivation procedure tested in this study requires that all component algorithms locally available for the identification of the condition of interest should be extracted, tested and stored regardless of whether they will be subsequently included in the final recommended composite algorithm. This also gives to investigators and local experts the chance to tweak the preferred identification algorithm at the study design stage, according to the study questions.

### Gaining insight into cases identified by data source-tailored case-finding algorithms

In this study, the composite algorithms recommended by local experts for the identification of T2DM were extremely variable, resulting, however, in a selection of cases that are likely to represent the best possible local approximation of the true case identification. Indeed, since the age-specific prevalence of diabetes is expected to be fairly homogeneous across the geographic areas we are considering,[29] the observed differences in terms of percentage of the corresponding study populations can be interpreted in light of both the specific components adopted and of relevant data sources’ characteristics. Among all data sources, the highest percentage of cases was identified in HD because this data source only captures subjects who visit the hospital, who, by definition, will have a higher burden of disease with respect to the general population. On the other extreme, the BD showed the lowest percentage, possibly because people volunteering to participate in this data source are slightly healthier than the general population. Both HD and BD identify patients with T2DM using DIAG-based component only. However, while in HD cases were identified among inpatients only who are expected to be at a more advanced stage of the disease and more likely to have comorbidities,[5] in BD characteristics of cases were probably more representative of patients with T2DM in the corresponding source population, because diagnoses are recorded in a primary care setting. As for the three primary care data sources, the Italian PCD adopted a case finding strategy based on data from DIAG and LABVAL. This strategy was expected to be very sensitive. Moreover, in a previous validation study, it was also proven to have the highest possible PPV. [27] Therefore, its recommended algorithm can be considered an excellent approximation of a true case identification and the observed percentage of cases can be assumed to be a valid estimate of the prevalence of T2DM in the correspondent source population. In the PCD from UK a lower percentage of cases was identified compared to the Italian PCD. This result could be due to a slight underreporting of diagnoses in the data source. As for the Dutch PCD, the age-specific percentage of detected cases was almost identical to that observed in the PCD from UK. However, in the Dutch PCD a DRUG-based algorithm was adopted as additional inclusion criterion to the DIAG-based component DIAG_T2DM_PC, since the latter was not sensitive enough when used alone. In fact, general practitioners participating to the Dutch PCD often record diagnoses using free text description which may sometimes remain elusive to the keywords-based retrieval process. Among RLDs, the percentage of the population identified in the Dutch RLD was slightly lower than that observed in the other two RLDs from Italy and Denmark respectively. Indeed, local experts of the Dutch RLD recommended the use of one single DRUG-based component (DRUG_ORAL) as the preferred case-finding algorithm, while the other two data sources, on the grounds of previous validation studies,[25, 28] adopted more complex composite algorithms that allowed to increase sensitivity by including also components based on DIAG and/or TEST. In particular, the Danish RLD was the only data source collecting diagnoses from secondary care. Notably, TEST-based components, which identify patients through specific patterns of utilization of glycated haemoglobin tests, were not included in the Italian RLD since they resulted to be far more unspecific than in the Danish RLD. This was clearly showed when the impact of TEST-based components on the total population of cases identified in the two data sources was observed. Such a difference was probably due to local healthcare system organization and guidelines with respect to diagnosis and follow-up of diabetic patients.

### Understanding quality of a local case-finding algorithm

In studies utilizing routinely collected health data, understanding the quality of local case-finding algorithms is paramount for the interpretation of study findings[11, 30] and *a fortiori* in multi-data source studies. The component algorithm strategy proposed in this study can indirectly provide approximation of algorithm validity indexes, even when no formal validation studies are available for one or more of the participating data sources. This is attained through the benchmarking of components and composite algorithms across data sources with similar characteristics but collecting data from different geographic areas or *vice versa*.

Indeed, in this study, cases in PCDs were basically identified through primary care diagnoses and are thus expected to be fairly representative of the T2DM patients in the corresponding source populations. In RLDs, instead, most of cases were captured through non-insulin antidiabetic drugs utilization which cannot identify those patients at a earlier stage of the disease who are not on drug treatment (do diet only) and may also misclassify T2DM with other diseases for which the same drugs can sometimes be used (e.g. polycystic ovary syndrome).[4] Supposing that the validity of the latter case-finding algorithm was completely unknown, data reported in Table 3 can be used to obtain an approximation of its expected sensitivity and PPV. As an example, the Dutch RLD, which used a case-finding algorithm based on the utilization of non-insulin antidiabetics only (i.e. DRUG_ORAL) can be considered. Since sensitivity corresponds to the percentage of subjects with a true diagnosis of T2DM who also have the DRUG_ORAL pattern of non-insulin antidiabetic drugs utilization, this percentage can be estimated from the Dutch PCD to be around 77%, or slightly lower if we accept that sensitivity in the Dutch PCD is not 100% (the corresponding percentage in the other two PCD data sources is lower than 55%). PPV, instead, is the percentage of subjects utilizing oral antidiabetics who really have type 2 diabetes. In this case, value higher than 90% is expected since other indications for such drugs have a very low prevalence.[4] In fact, this is also confirmed in both PCDs from Italy and UK where the component DRUG_ORAL added less than 3% of cases when used as additional inclusion criteria.

### Tailoring selection of components to a research question

Since this study was solely intended as an exercise to test the feasibility of the methodology proposed, the research question was rather generic and, consequently, not all the composite algorithms were chosen with the primary objective of addressing specificity or sensitivity. In general, the preferences of local experts were more often directed towards sensitivity, at expense of specificity, with the notable exception of RLD-NL. Diagnosis-based components were selected preferentially because of their face validity. Components based on unspecified type of diabetes (DIAG_DMUNSPEC or DIAB_OTH) were used in PCD-I and RLD-I due to specific characteristics of the local data source. Moreover, components that minimally or slightly increased capture were generally included, while those which were not specific were dropped if they inflated capture, e.g DRUG_INSULIN and DRUG_ORAL in PCD-UK, in order to avoid misclassification with type 1 with type 2 diabetes.

Indeed, at the design stage of a specific study, the proposed data derivation procedure allows investigators and local experts to modify their preferred identification algorithm according to the type of study question or sensitivity analysis. In the case of a study involving T2DM, if specificity is important, they may switch to DIAG-based components at the expenses of sensitivity. This may happen, for instance, when studying occurrence of organ complications in patients with T2DM. In case sensitivity is important, they may add other inclusion criteria, like TEST-based components: this may be recommended in safety studies. Finally, if homogeneity across different data sources is important, investigators and local experts may agree to adopt a DRUG-based strategy.

### Limitations

Although this was a proof-of-concept study in which results obtained were not intended as estimates of disease frequencies, limitations that might have biased the results and comparisons discussed in this manuscript must be acknowledged. In particular, formal validation of the retrieved cases against medical chart review was not performed as well as important variables other than age were not considered for stratification of results. Finally, identifying T2DM with an algorithm that is not validated represents a limitation because the estimates of validity indices rely on the subjective judgment of the local expert. Nevertheless, in a multi-national, muti-data source study, in absence of a previously validated algorithm, the local expert recommendation remained the best possible choice.

## Conclusions

Through the identification of T2DM cases, this study demonstrates that the standard procedure for data derivation developed within the EMIF project represent a methodological advancement for the execution of multi-national, multi-data source studies. In fact, on the basis of a shared definition of any event of interest, the procedure assures interoperability of heterogeneous EHR systems and allows establishing data-source tailored case-identification algorithm in a standardized fashion, providing sufficient information for contextualization and correct interpretation of study results and generating transparent and reusable documentation on the entire data derivation process. Further studies are warranted to explore the validity of different components and composite algorithms as well as the heterogeneity of the population identified across data sources.

## Supporting Information

S1 Fig Comparison of results from individual component algorithms. (PDF)

S1 Table Mapping of local codes and free text keywords corresponding to the medical concepts embedded in the component algorithms adopted for type 2 diabetes identification. (DOC)
