# Supplementary material for: Identifying Cases of Type 2 Diabetes in Heterogeneous Data Sources: Strategy from the EMIF Project
Source: PLoS One. 2016 Aug 31;11(8):e0160648. doi: 10.1371/journal.pone.0160648 (PMC5006970; doi:10.1371/journal.pone.0160648)

a) Component algorithms based on records of diagnoses

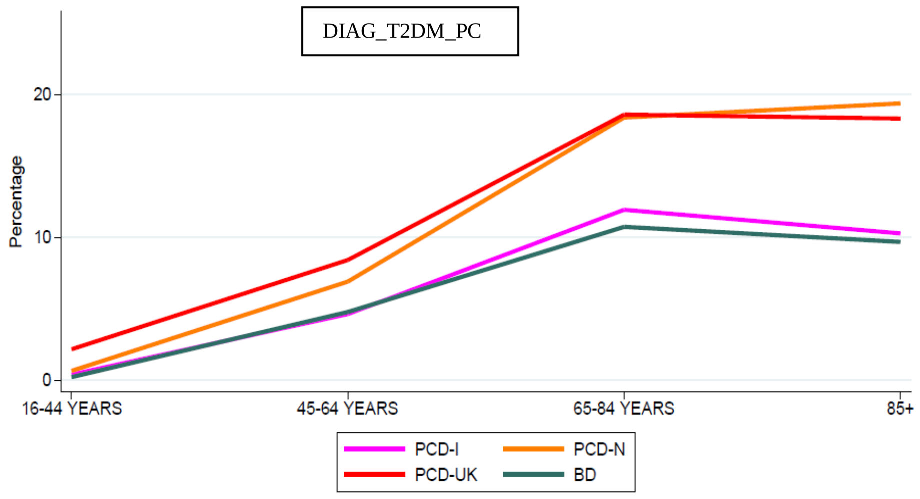

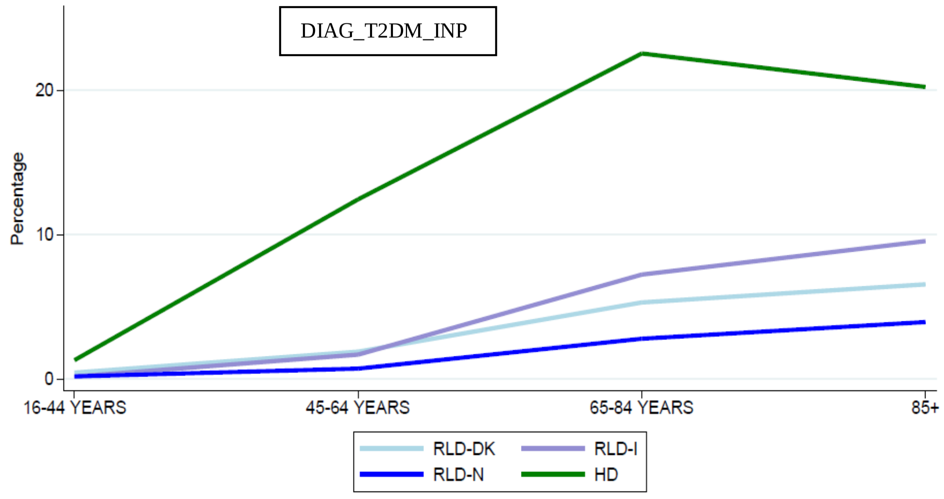

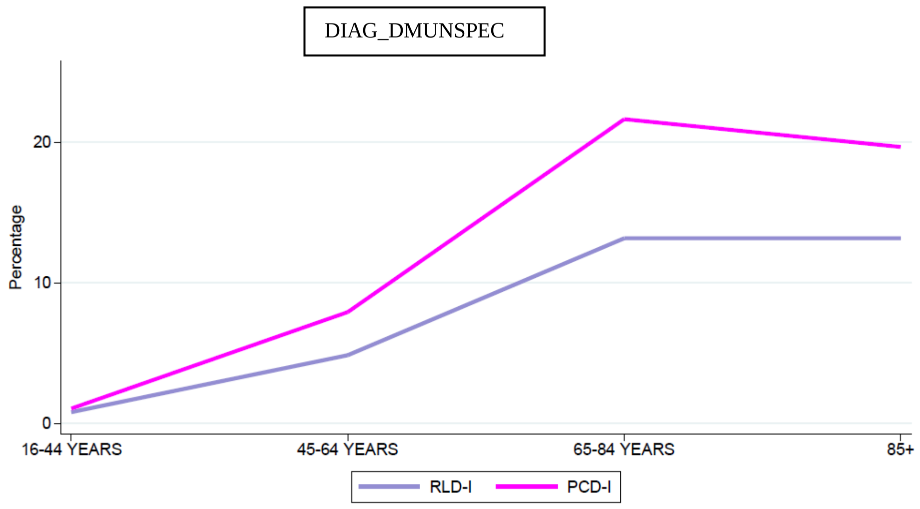

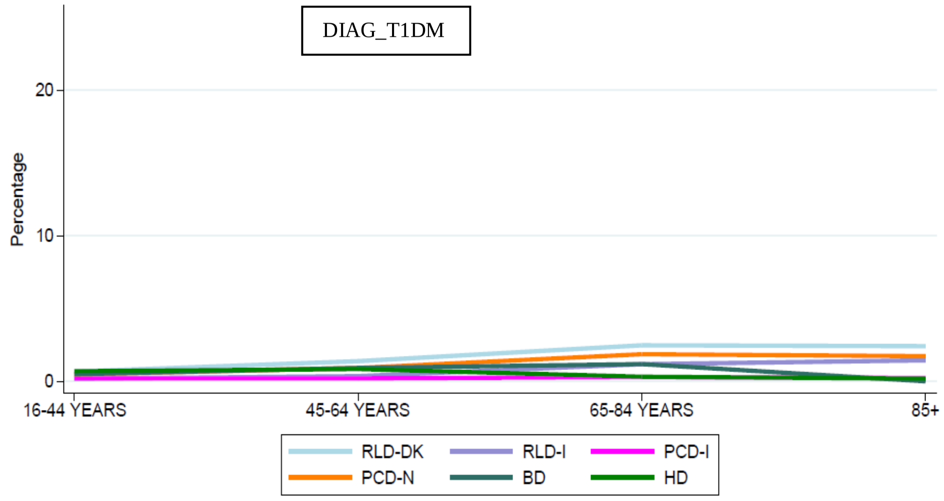

b) Component algorithms based on records of drug utilization

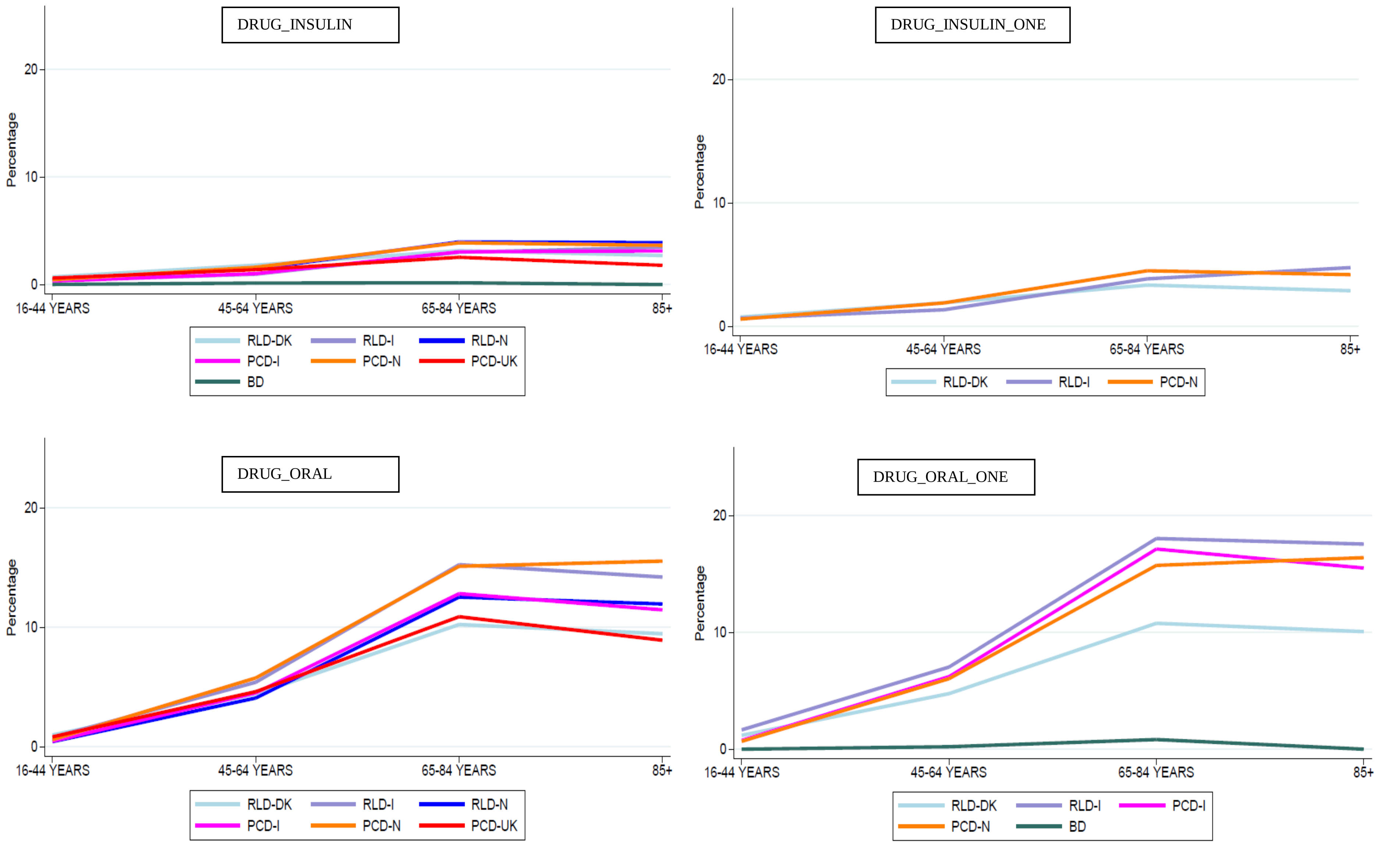

c) Component algorithms based on records of utilization of diagnostic tests

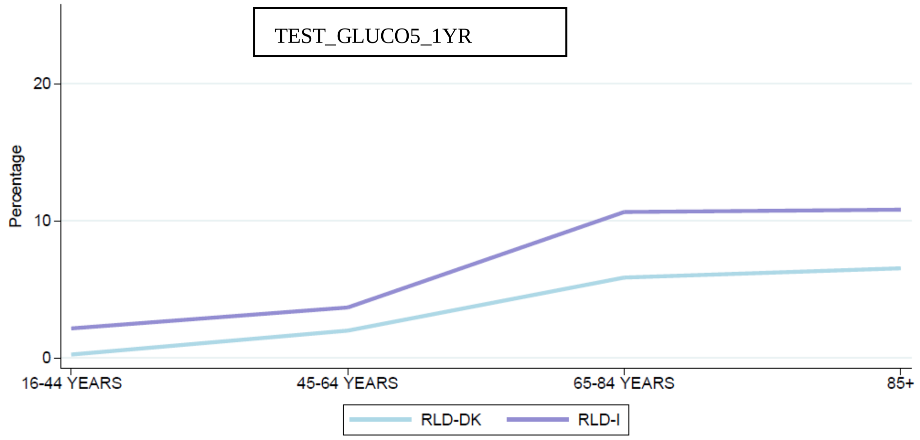

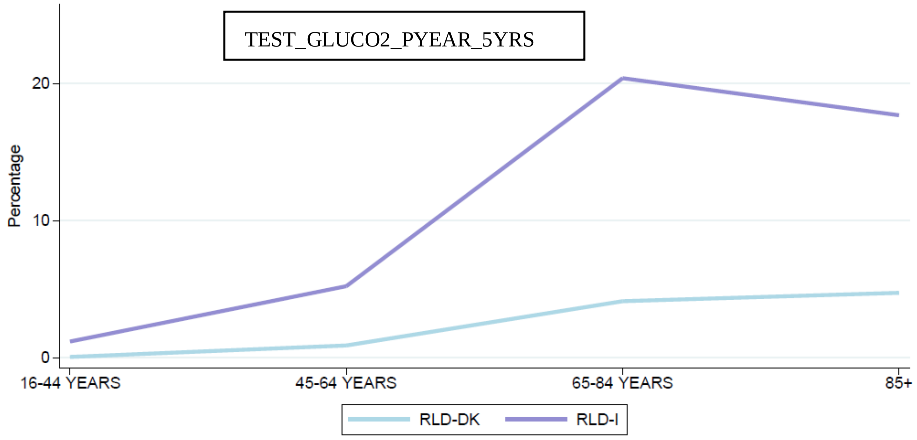

d) Component algorithms based on records of laboratory results

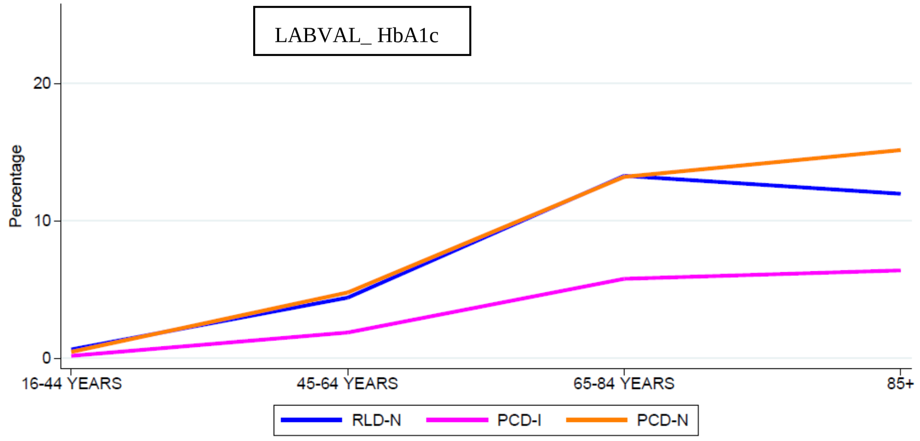

Supplement: S1 Fig — (PDF) [file pone.0160648.s001.pdf]
