# Supplementary material for: Identifying Cases of Type 2 Diabetes in Heterogeneous Data Sources: Strategy from the EMIF Project
Source: PLoS One. 2016 Aug 31;11(8):e0160648. doi: 10.1371/journal.pone.0160648 (PMC5006970; doi:10.1371/journal.pone.0160648)
Supplement: S1 Table — (DOC) [file pone.0160648.s002.doc]

**Table 1S.** Mapping of local codes and free text keywords corresponding to the medical concepts embedded in the component algorithms adopted for type 2 diabetes identification.

**- Diabetes mellitus type I**

|  | **Local Terminology** | | | | |
| --- | --- | --- | --- | --- | --- |
| **CUI^a^** | **ICD9CM** | **ICD10** | **ICPC** | **RCD** | **Free text used by RLD-N^b^** |
| C0375131 | 250.41 |  |  |  |  |
| C0375118 | 250.11 |  |  |  |  |
| C0375148 | 250.83 |  |  |  |  |
| C0375116 | 250.03 |  |  |  |  |
| C0375127 | 250.31 |  |  |  |  |
| C0011854 |  | E10 | T89001  T89002  T89003  T90004  T90006  T90008 | X40J4 |  |
| C0375123 | 250.21 |  |  |  |  |
| C0375146 | 250.81 |  |  |  |  |
| C0342302 |  |  |  | 66AJ1 |  |
| C0375150 | 250.91 |  |  |  |  |
| C0375135 | 250.51 |  |  |  |  |
| C0375125 | 250.23 |  |  |  |  |
| C0375133 | 250.43 |  |  |  |  |
| C0375129 | 250.33 |  |  |  |  |
| C0375152 | 250.93 |  |  |  |  |
| C0375114 | 250.01 |  |  |  |  |
| C0375136 | 250.53 |  |  |  |  |
| C0375120 | 250.13 |  |  |  |  |
| C0375138 | 250.61 |  |  |  |  |
| C0011855 |  |  |  |  | ('T90') AND  ('type 1') |
| C0375142 | 250.71 |  |  |  |  |
| C0375144 | 250.73 |  |  |  |  |
| C0375140 | 250.63 |  |  |  |  |

^a^Concept unique identified from the Unified Medical Language System (UMLS).

^b^RLD-N: record linkage data source from Netherlands.

**- Diabetes type 2**

|  | **Local Terminology** | | | | |
| --- | --- | --- | --- | --- | --- |
| **CUI^a^** | **ICD9CM** | **ICD10** | **ICPC** | **RCD** | **Free text used by RLD-N^b^** |
| C0375151 | 250.92 |  |  |  |  |
| C0011860 |  | E11 | T90005  T90007  T90009 | X40J5 | ('T90') AND  ('type 2') |
| C0375143 | 250.72 |  |  |  |  |
| C0375117 | 250.10 |  |  |  |  |
| C0375126 | 250.30 |  |  |  |  |
| C0375149 | 250.90 |  |  |  |  |
| C0375147 | 250.82 |  |  |  |  |
| C0375141 | 250.70 |  |  |  |  |
| C0375119 | 250.12 |  |  |  |  |
| C0375122 | 250.20 |  |  |  |  |
| C0375132 | 250.42 |  |  |  |  |
| C0375130 | 250.40 |  |  |  |  |
| C0375134 | 250.50 |  |  |  |  |
| C0375145 | 250.80 |  |  |  |  |
| C0375115 | 250.02 |  |  |  |  |
| C0375113 | 250.00 |  |  |  |  |
| C0376128 | 250.52 |  |  |  |  |
| C0375137 | 250.60 |  |  |  |  |
| C0375124 | 250.22 |  |  |  |  |
| C0375128 | 250.32 |  |  |  |  |
| C0375139 | 250.62 |  |  |  |  |

^a^Concept unique identified from the Unified Medical Language System (UMLS).

^b^RLD-N: record linkage data source from Netherlands.

**- Diabetes unspecified**

|  | **Local terminology** | | | | |
| --- | --- | --- | --- | --- | --- |
| **CUI^a^** | **ICD9CM** | **ICD10** | **ICPC** | **RCD** | **Exemption*** |
| C0011884 | 362.0 | H36.0 | F83002 | F420.  F420z  XaBul |  |
| C0011881 | 250.4 |  | U88011 | K01x1  X30Kk |  |
| C0011880 | 250.1 |  |  | C101.  C101z |  |
| C0021645 |  |  | T87002 | X40Jo |  |
| C0341893 | 648.0 648.00 |  |  | L180.  L1803  L180z |  |
| C0011850 |  |  |  |  | 250 |
| C0011882 | 250.6 |  | N94012 | X00Ag |  |
| C0032969 | 648.03 | O24  O24.9 |  |  |  |
| C0341897 | 648.01 |  |  | L1801 |  |
| C0271635 | 250.0 | E14.9 |  | C100.  C100z |  |
| C0154830 | 362.02 |  |  | F4201 |  |
| C0154183 | 250.8 |  |  |  |  |
| C0271680 | 357.2 | G63.2 |  | XE15k |  |
| C0011849 | 250 | E10-E14.9  E14 | T90002 | C10.. |  |
| C0341896 | 648.02 |  |  | L1802 |  |
| C0011871 | 250.7 | E14.5 |  |  |  |
| C0342245 | 250.5 |  |  | C105.  C105z |  |
| C0342257 | 250.9 | E14.8 |  | C10z.  C10zz |  |
| C0011876 | 366.41 | H28.0 |  | F4640 |  |
| C0375121 | 250.2 |  |  |  |  |
| C0043207 |  |  |  | X008t |  |
| C1260459 | V45.85 |  |  |  |  |
| C1260460 | V53.91 |  |  |  |  |
| C1260472 | V65.46 |  |  |  |  |
| C1455979 | V58.67 |  |  |  |  |

^a^Concept unique identified from the Unified Medical Language System (UMLS).

*Italian classification of diseases for which patients are exempt from copayment of related healthcare services.

**- Metabolic problems around pregnancy**

|  | **Local terminology** | | | |
| --- | --- | --- | --- | --- |
| **CUI^a^** | **ICD9CM** | **ICD10** | **ICPC** | **RCD** |
| C0156878 | 648.8 |  |  |  |
| C0085207 |  | O24.4 | W77004  W85001 | L1808 |

^a^Concept unique identified from the Unified Medical Language System (UMLS).

**- Metabolic/pancreatic problems, non type 2 diabetes**

|  | **Local terminology** | | | |
| --- | --- | --- | --- | --- |
| **CUI^a^** | **ICD9CM** | **ICD10** | **ICPC** | **RCD** |
| C0161508 | 962.0 |  |  | SL20.  SL20z |
| C0154192 | 251.8 | E16.8 |  | C11y.  Cyu31 |
| C0524620 | 277.7 |  |  | X40JE |
| C0271640 | 249 |  |  | X40JA |

^a^Concept unique identified from the Unified Medical Language System (UMLS).

**- Polycystic ovary syndrome**

|  | **Local terminology** | | | |
| --- | --- | --- | --- | --- |
| **CUI^a^** | **ICD9CM** | **ICD10** | **ICPC** | **RCD** |
| C0032460 | 256.4 | E28.2 | T99040  T99043 | X406n  X40NO  XE10l |

^a^Concept unique identified from the Unified Medical Language System (UMLS).

**- Drugs used in diabetes, excl insulin**

|  | **Local terminology** |
| --- | --- |
| **CUI^a^** | **ATC** |
| C3653344 | A10B |

^a^Concept unique identified from the Unified Medical Language System (UMLS).

**- Insulins and analogues**

|  | **Local terminology** |
| --- | --- |
| **CUI^a^** | **ATC** |
| C3653579 | A10A |

^a^Concept unique identified of the Unified Medical Language System (UMLS).
